# Impact of uterine closure techniques following cesarean section on the development of cesarean scar defects: consigning cicatrix to history?

**DOI:** 10.1590/1806-9282.20250706

**Published:** 2026-06-19

**Authors:** Ilker Sengul, Demet Sengul

**Affiliations:** 1 Giresun University, Faculty of Medicine, Division of Endocrine Surgery – Giresun, Turkey.; 2 Giresun University, Faculty of Medicine, Department of General Surgery – Giresun, Turkey.; 3 Giresun University, Faculty of Medicine, Department of Pathology – Giresun, Turkey.

Dear Editor,

Cesarean section (C/S) scar defect (CSD), per se, has always occupied minds for a considerable time, which requires a graceful approach^1-7^. I am writing to you as an appraisal of the article entitled "Is cesarean scar defect becoming history? The effect of uterotomy closure" by Gumusburun and Uskent^8^, published in volume 71, Rev Assoc Med Bras. This article presents a significant investigation into the impact of uterine closure techniques following C/S on the development of CSD, also known as isthmocele pathology. The primary strength of this study lies in its novel investigation of the "baseball" type suture technique in the context of isthmocele formation. Gumusburun and Uskent specifically evaluated the effects of this technique, comparing traditional single-layer locked and unlocked continuous suturing methods. The study's prospective design and the direct comparison of three distinct surgical techniques during primary C/S also contribute to its methodological rigor. The findings of the study are compelling, suggesting that the baseball-type closure significantly attenuated its incidence and led to a greater residual myometrial thickness (RMT) at the cicatrix site compared to the single-locked and single-unlocked techniques. Notably, isthmocele development was considerably lower in the baseball group compared to the single-locked and -unlocked ones. Furthermore, the highest mean RMT was observed in the baseball suture group, whose outcomes underscore the potential importance of the uterotomy closure technique in influencing cicatrix healing and preventing its formation. The authors rightly conclude that baseball-type suturing appears to be an advantageous method for preserving RMT and preventing isthmocele^8^. However, it is important to kindly acknowledge certain limitations of this study. The authors themselves mention that the statistical power was limited to 80%, and the postoperative sonographic evaluations were conducted only at the third month postpartum. While these findings are valuable, further studies with a higher power (e.g., 90%) and longer-term follow-up are needed to assess the long-term complications associated with that and to strengthen these initial observations. Moreover, the inclusion criteria for the study were quite restrictive, including only term pregnancies (>37 weeks) with no prior C/S, no active labor exceeding 4 cm cervical dilatation, and specific body mass index limits (<35 kg/m²). The exclusion of women with factors such as placental abnormalities, previous uterine surgery, multiple pregnancies, and certain comorbidities limits the generalizability of the findings to the broader population undergoing C/S. In fine, while this work meticulously details the three suture techniques used, it is noted that only one type of suture material was used across all groups. Therefore, future research could explore the potential impact of different suture materials on isthmocele formation. As a matter of fact, this study provides valuable initial evidence suggesting the superiority of the baseball-type uterine closure technique in reducing isthmocele pathology and improving RMT. This research recommends a promising avenue for refining surgical techniques during C/S to potentially mitigate the increasing prevalence of CSD and its associated complications. However, the identified limitations necessitate further investigation through larger, multicenter studies with long-term follow-up to validate these findings and to assess their applicability across a broader range of cases undergoing Cesarean delivery. This study serves as an important foundation for future research aimed at optimizing uterotomy closure techniques and potentially providing cesarean cicatrix defects, a concern of the past. This issue merits further investigation. We thank Gumusburun and Uskent^8^ for their valued study of uterotomy closure techniques in order to completely prevent CSD and consign it to history.

## Data Availability

The datasets generated and/or analyzed during the current study are available from the corresponding author upon reasonable request.
